# Microfluidic co-culture system for cancer migratory analysis and anti-metastatic drugs screening

**DOI:** 10.1038/srep35544

**Published:** 2016-10-20

**Authors:** Shengli Mi, Zhichang Du, Yuanyuan Xu, Zhengjie Wu, Xiang Qian, Min Zhang, Wei Sun

**Affiliations:** 1 Biomanufacturing Engineering Laboratory, Advanced Manufacturing Division, Graduate School at Shenzhen, Tsinghua University, Shenzhen, P. R. China; 2 Open FIESTA Center, Tsinghua University, Shenzhen 518055, P. R. China; 3 Department of Mechanical Engineering and Mechanics, Tsinghua University, Beijing, China; 4 Department of Mechanical Engineering, Drexel University, Philadelphia, PA, USA; 5 Tsinghua-Berkeley Shenzhen Institute, Shenzhen, P. R. China

## Abstract

Tumour metastasis is an important reason for cancer death, and cancer cell migration is an important step in the process of tumour metastasis. Studying cancer cell migration is of great significance. Here, we present a novel microfluidic co-culture system and establish mild, moderate and severe cancer models by using HMEpiC and MDA-MB–231 cells to study cancer cell migration and anti-cancer drug screening. Using this device, we achieved high cell viability (over 90%) and a stable analysis of the migration ability of cancer cells. We observed that the density of the cancer cells determined the probability of the occurrence of metastatic cells and that the induction of normal cells affected the metastatic velocity of each cancer cell. We verified that the increase in the migration ability of MDA-MB-231 cells co-cultured with HMEpiC cells was relative to the increased secretion of IL-6 and that this was verified by an IL-6 inhibitor assay. This co-culture also led to decreased CK-14 secretion and morphological changes in HMEpiC cells. Finally, significant inhibition of paclitaxel and tamoxifen on cancer migration was observed. Taken together, our microfluidic device could be a useful tool for the quantitation of the migratory capability and anti-metastatic drug screening.

Cancer is a serious human health problem worldwide^1^,^2^, and metastasis is responsible for as much as 90% of cancer-associated mortality, yet it remains the most poorly understood component of cancer pathogenesis^3^. Breast tumours represent the most frequently diagnosed cancer in women and are also the leading cause of cancer-related death among the female population^4^,^5^. Thus, it is very necessary to study the migration of breast cancer and to develop effective anti-cancer drugs, especially anti-metastatic drugs.

The tumour microenvironment is a critical component of cancer biology and is responsible for metastasis and drug resistance^6^,^7^,^8^,^9^. The migration of cancer cells is maintained by the dynamic interplay between the tumour cells and many distinct cell types that exist in the adjacent microenvironment, including endothelial cells, fibroblasts, and so on^10^,^11^,^12^,^13^,^14^,^15^. The construction of a multicellular co-culture system that mimics the breast tumour microenvironment is very important for investigating the interaction of cancer cells and non-malignant cells and the role of non-malignant cells in the progression of cancer cell migration. Traditional *in vitro* models for studying cell migration, such as transwell and would healing assays, often lack real-time information on migration dynamics, require a large number of sample cells and are unable to accurately quantify the migratory capability at different cells in the environment^16^,^17^. These approaches for assessing *in vitro* breast cancer cell migration often are based on monoculture, and they do not simulate the conditions of the human environment well^18^,^19^,^20^. Therefore, the biggest need for breast cancer migration research is still to reconstitute a more bionic tumour environment and to establish a more feasible and high-throughput evaluation system for cancer cell migration.

In the past decade, microfluidic technology with evident advantages, such as small sample volume, high sensitivity, fast processing speed, high portability and low cost, has become an increasingly promising tool for basic and applied research on cancer^21^,^22^,^23^. The use of microfluidic chips can better mimic the tumour microenvironment for studying cell migration and anticancer drug screening. For example, Zhang and co-workers developed a high-throughput device, the M-Chip, to investigate the mesenchymal mode of breast cancer cell migration^16^. Nguyen *et al*. developed an impedance sensing-based microfluidic device to investigate the migration of breast cancer cells embedded in 3D matrices^24^. Haessler *et al*. developed a microfluidic device to study how fluid flow in tumour microenvironments affects cancer cell migration^25^. Choi *et al*. described a micro-engineered pathophysiological model to test anticancer drugs in a breast cancer-on-a-chip device^10^. Chen *et al*. developed a microfluidic chip for mimicking the physiological microenvironment of solid tumours with multicellular tumour spheroids for anticancer drug screening^26^.

Although there are many studies regarding breast cancer migration based on microfluidic devices, and some scholars have established co-culture tumour models in microfluidic devices to solely study cancer cell migration^11^,^27^, only a few studies have focused on the interaction between cells and studied the role of normal cells in breast cancer migration^10^,^28^. In addition, the factors of the tumour microenvironment, such as cell density, greatly influence cancer cell migration and drug screening^10^,^25^,^26^,^29^,^30^. In different regions of a metastatic breast tumour (Fig. 1(a)), the densities of cancer cells and non-malignant cells are different, and the migration ability of cancer cells in these regions may be not the same. However, few researchers have studied the effect of the cell density of both cancer cells and non-malignant cells on cancer cell migration in microfluidic devices.

Here, we present a novel microfluidic system to establish an *in vitro* co-culture model that mimics different regions of a metastatic breast tumour to study cancer cell migration and anti-cancer drug screening. The microfluidic chip contains three groups of co-culture chambers with microchannel arrays for the detection of cancer cell migration and with fluid channels for the delivery of nutrients and anticancer drugs. By controlling the densities of the normal breast cells HMEpiC and the breast cancer cells MDA-MB–231 in the co-culture chambers, a mild cancer model, a moderate cancer model and a severe cancer model were established. Using the microfluidic chip, we first studied the viability of cells on the chips. Then, by transfecting the HMEpiC cells with RFP (red fluorescent protein) markers and the MDA-MB–231 cells with GFP (green fluorescent protein) markers, we compared the migration ability of the cancer cells in the three cancer models. Through immunofluorescence staining and migration tests, we analysed the interaction between the HMEpiC and MDA-MB–231 cells. Finally, by adding different concentrations of paclitaxel and tamoxifen, we studied the effect of the drugs on cancer cell migration. In summary, this microfluidic system provides a novel way to mimic the *in vivo* tumour microenvironment, which can be used to perform anti-metastatic drug screening at different cell densities in breast tumours.

## Materials and Methods

### Device design and fabrication

**Figure 1:**
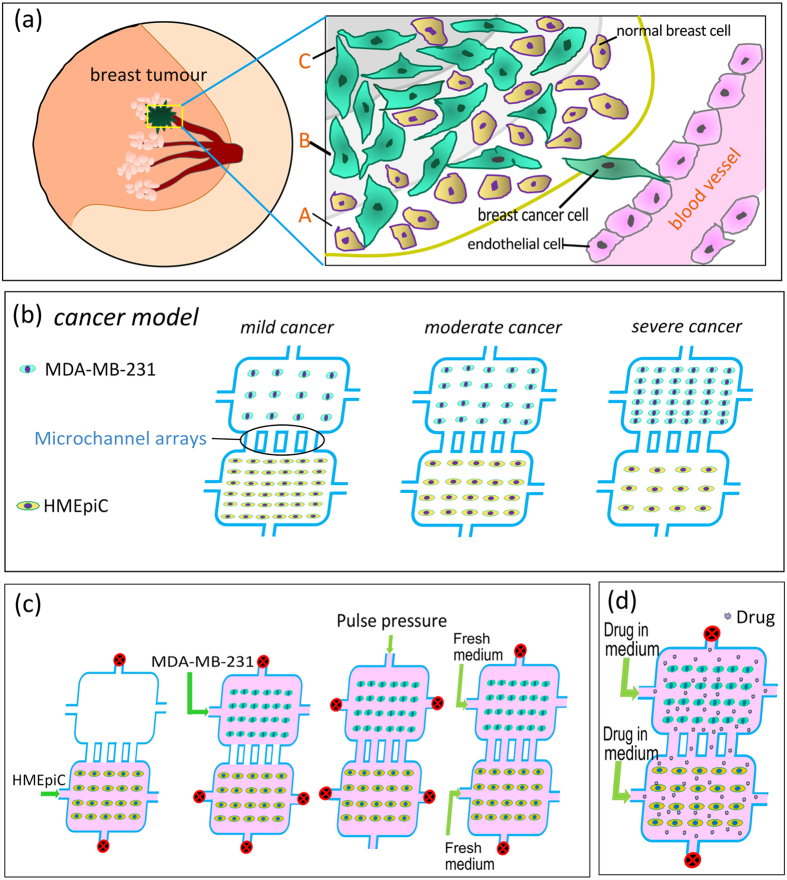
(**a**) Schematic illustration of the metastatic breast tumour. (**b**) Schematic illustration of three cancer models. (**c**) The steps for loading the cells and the realization of co-culture. (**d**) Drug treatment on chip.

Near the centre of a metastatic breast tumour, cancer cells are denser and normal cells are less dense (Fig. 1(a)). By controlling the densities of cancer cells and non-malignant cells, we established mild, moderate and severe cancer models in co-culture chambers (Fig. 1(b)). In our design, each co-culture chamber had two cell culture chambers: one for the cancer cell culture and another for the normal cell culture. The two chambers in each cancer model were connected by unified microchannel arrays, which were designed to analyse cancer cell migration.

**Figure 2:**
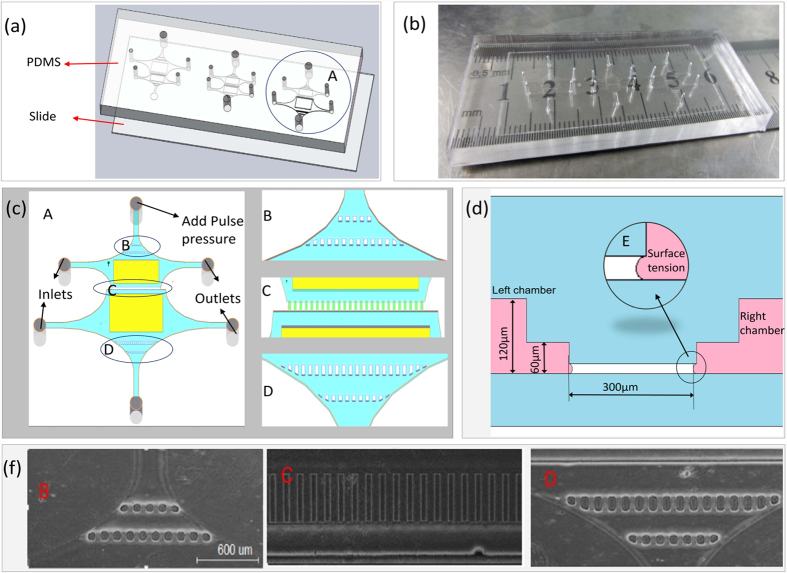
(**a**) Design of microfluidic co-culture devices. (**b**) A picture of the microfluidic devices. (**c**) Schematic illustration of co-culture chamber A. (**d**) A sectional view of microchannel arrays C. (**f**) A optical image of positions B, C and D, scale bar: 600 μm.

The microfluidic devices were fabricated using standard soft-lithography techniques with replica moulding poly (dimethylsiloxane) (PDMS), as previously described^31^,^32^,^33^,^34^. The microfluidic chip was composed of a glass slide layer and a PDMS layer with the same width (2.7 cm) and length (6.9 cm) (Fig. 2(a)) and a different thickness (0.2 mm and 6 mm, respectively). The PDMS layer had three co-culture chambers, each with two culture chambers and the same microchannel arrays. The area of the normal cell culture chamber in the mild cancer model and the area of the cancer cell culture chamber in the severe cancer model were twice the area of the last four chambers (Fig. 2(a)), and this design can guarantee the uniformity of cell density in the zone near the microchannel arrays when seeding a high density of cells. The microchannel arrays had 27 microchannels (60 μm wide, 300 μm long, and 10 μm high) (Fig. 2(c,f), area C). Each co-culture chamber had six holes, four holes for cell loading and cell medium supplementing and two holes for loading a pulse pressure. Two culture chambers of a co-culture chamber were separated by microchannel arrays due to surface tension^35^ and were connected by loading a pulse pressure (Fig. 2(d)). The flow channels were 60 μm high, and the cell culture channels in Fig. 2(c) in the yellow area were 120 μm high. The microbump arrays B and D shown in Fig. 2(c) were designed to guarantee the uniformity of the hydraulic pressure and to reduce the influence of the pulse pressure on the cells.

### Cell culture and the formation of the on chip cancer models

MDA-MB-231 breast cancer cells from ATCC were cultured in Leibovitz L 15 medium (Life Technologies Corporation) supplemented with 10% foetal bovine serum, penicillin (100 units/ml) and streptomycin (100 μg/ml). Mammary epithelial cell medium (MECM, ScienCell) for the human mammary epithelial cells (HMEpiC, ScienCell) consisted of 500 ml of basic medium, 5 ml of mammary epithelial cell growth supplement (MEpiCGS, ScienCell) and 5 ml of penicillin/streptomycin (P/S). The MDA-MB-231 and HMEpiC cells were both cultured in a cell incubator with 5% CO_2_ at 37 °C.

Before loading the cells, we pre-treated the microfluidic devices with 75% alcohol for 12 h to disinfect and remove air bubbles in the co-culture chamber, UV sterilised the devices for 30 minutes and coated the cell chambers with 1 mg ml^−1^ poly-L-lysine (PLL) (Sigma-Aldrich) in 0.1 M borate buffer (pH 8.5) for 12 h at 37 °C. Then, the cell chambers were equilibrated with cell medium for 30 min.

The cell suspensions of two types of cells were manually loaded into the cell inlets of the chip (Fig. 1(c)). The HMEpiC cells at densities of 6 × 10^6^ cells ml^−1^, 2 × 10^6^ cells ml^−1^ and 0.5 × 10^6^ cells ml^−1^ and the MDA-MB-231 cells at densities of 0.5 × 10^6^ cells ml^−1^, 2 × 10^6^ cells ml^−1^ and 6 × 10^6^ cells ml^−1^ were loaded into the co-culture chambers of the mild, moderate and severe cancer models, respectively. Due to the surface tension of the microchannel arrays, the cell suspensions filled the cell culture channels without getting into the microchannels. After 6 h, we added fresh cell culture medium to wash out the non-adherent cells, and then we plugged the cell inlets and outlets, as shown in Fig. 2(c), and added a pulse pressure to one of the last two holes to establish a true co-culture.

### Cell viability analysis in the chip

We used the Live/Dead Cell Imaging Kit (Molecular Probes, Life Technologies Corporation) to evaluate cell viability in our models. In the Live/Dead assay, the viable cells are stained green, while the dead cells are stained red^35^. The cells were loaded into the microfluidic devices, as described previously, and after one day, we used phosphate buffered saline (PBS, HyClone) to wash the culture chambers in the microfluidic chip for 1–3 min. Then, the cells were incubated with the Live/Dead Cell Imaging Kit for 15–30 min at 37 °C. Next, we used PBS again to wash out the reagent for 3–5 min and observed the culture chambers under a fluorescent microscope. Finally, cell viability was measured by assessing the percentage of green fluorescent cells in all of the cells.

### Generation of fluorescent protein expressing cells and cell migration evaluation

In order to facilitate the observation of cancer cell migration, GFP and RFP (mCherry cDNAs) were used as tracer markers for the transfection of MDA-MB–231 and HMEpiC cells, respectively. The cells were plated onto a 6 cm petri dish, and 12–16 hours later, we removed the culture medium and added a mixture of 1 ml of the GFP marker kit (or RFP marker kit for RFP transfection), 3 ml of fresh culture medium and 4 μl of polybrene (8 μg/ml). Then, at intervals of 15 to 30 min, we gently shook the petri dish. We added 4 ml of fresh culture medium to the petri dishes after 2 h and replaced the medium with fresh culture medium after 24 h. After 48 h, the results of the fluorescent transfection were observed. Then, the MDA-MB–231 and HMEpiC cells, with the GFP and RFP fluorescent markers, respectively, were loaded into the microfluidic devices, as described before, and cancer cell migration was observed every 24 h.

### Immunofluorescence staining and the interaction analysis between normal breast cells and breast cancer cells

Interleukin 6 (IL-6) is a pro-inflammatory cytokine shown to alter cell morphology and to modulate cell migration and the epithelial to mesenchymal transition (EMT)^36^,^37^. Cytokeratin 14 (CK-14) is a characteristic protein of epithelial cells and is highly expressed in HMEpiC cells. To study the interaction between MDA-MB-231 and HMEpiC cells, we cultured the cells in the microfluidic devices for 48 h with and without co-culture. Then, we used IL-6 antibody (ab6672, Abcam) against the IL-6 protein of MDA-MB-231 cells and cytokeratin 14 antibody (ab192056, Abcam) against CK-14 protein of HMEpiC cells. An immunofluorescence staining procedure was performed in the microfluidic platforms using standard immunocytochemistry techniques, as previously described^11^,^38^.

Additionally, cell migration tests of the cancer cells were performed with and without co-culture. In order to further study the role of IL-6 expression in cancer cells migration, IL-6 inhibition experiments on MDA-MB-231 cells were performed by 24 h of pre-incubation using IL-6 receptor antibody (IL-6 RA, ab47215, Abcam). After incubation, MDA-MB-231 cells were used for migration tests and immunofluorescence staining tests.

### Paclitaxel and tamoxifen treatments and the evaluation of cytotoxicity and anti-metastatic effect

For the drug tests, the anti-metastatic effects of paclitaxel (Sigma Aldrich) and tamoxifen (Sigma Aldrich) were studied. First, MDA-MB–231 and HMEpiC cells transfected with GFP and RFP, respectively, were loaded into four microfluidic devices, as described before, and 6 h later when the cells were adherent, we replaced the medium with fresh culture medium mixed with paclitaxel concentrations of 0.1 μM, 0.3 μM, 0.5 μM, or 1 μM or with tamoxifen concentrations of 5 μM, 10 μM, 30 μM, or 50 μM. Then, every 12 hours, we replaced the corresponding fresh culture medium mixed with drugs, and every 24 hours, we observed cancer cell migration.

### Cell imaging and image analysis

All of the cell images were obtained using a fluorescence microscope (Xcellence, Olympus). Image-Pro Plus 6.0 (Media Cybernetics, Silver Spring, MD) and SPSS V19.0 (SPSS Inc.) software were used to perform the image analysis and data statistical analysis. All of the experiments were performed in triplicate or quadruplicate, and the data are presented as the mean ± standard deviation (SD). A one-way analysis of variance (ANOVA) and Student’s t-tests were used for comparisons of each group. P-values less than 0.05 were considered statistically significant and are indicated with asterisks (*) and pound sign (#).

## Results

### Cell viability and realisation of the three cancer models in the microfluidic devices

**Figure 3:**
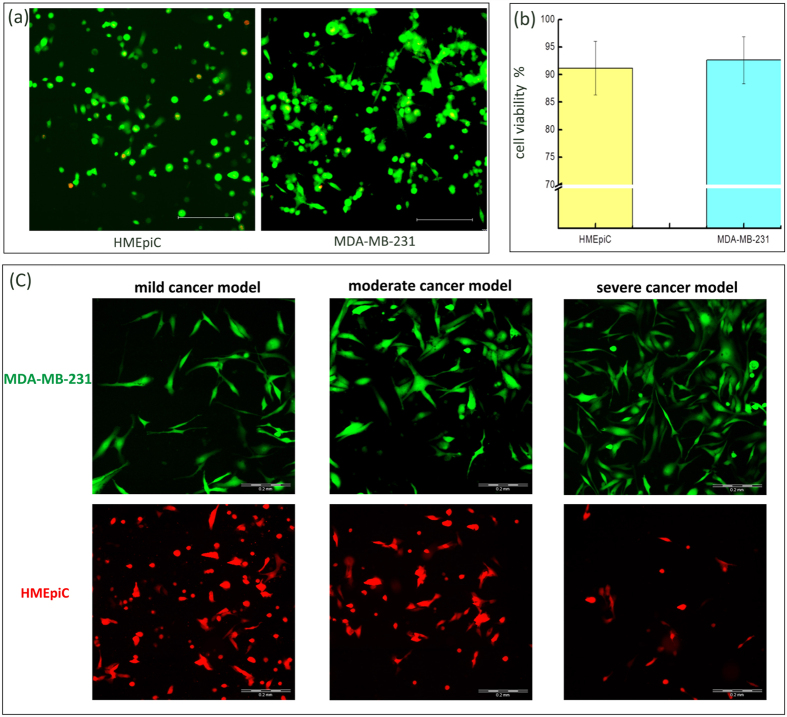
(**a**) Merged images of the live/dead staining images. (**b**) Cell viability of the MDA-MB-231 (92.6%) and HMEpiC (91.2%) cells, the error bars represent the standard deviations calculated from separate assays (n = 5). (**c**) Realisation of three cancer models. Scale bar of all images: 200 μm.

To quantify cell viability, three random domains per culture chamber were selected, and fluorescence images at wavelengths of 480 nm and 590 nm were captured. Subsequently, the green (live) and red (dead) cells were manually counted in the three random domains per sample. Finally, the data from three samples were statistically analysed using SPSS V19.0 software. The results are shown in Fig. 3(a,b), and the viability of both the MDA-MB-231 and HMEpiC cells were significantly above 90%.

In order to facilitate observation of the cells, GFP and RFP (mCherry cDNAs), as tracer markers, were used to transfect the MDA-MB–231 and HMEpiC cells, respectively. The HMEpiC cells, at densities of 6 × 10^6^ cells ml^−1^, 2 × 10^6^ cells ml^−1^ and 0.5 × 10^6^ cells ml^−1^, and the MDA-MB-231 cells, at densities of 0.5 × 10^6^ cells ml^−1^, 2 × 10^6^ cells ml^−1^ and 6 × 10^6^ cells ml^−1^, were loaded into the cell co-culture chambers of the mild cancer, the moderate cancer and the severe cancer models, respectively, as shown in Fig. 3(c). Images of the cells were taken 1 day after co-culture of the HMEpiC and MDA-MB-231 cells by fluorescence microscopy.

### Cancer cell migration evaluation in the microfluidic device

**Figure 4:**
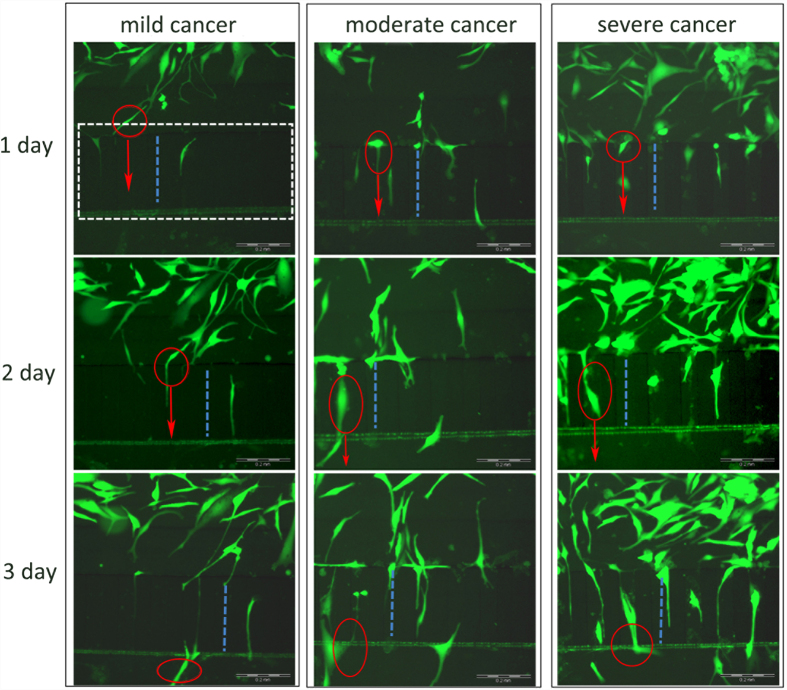
Migration images of the same location of each cancer model. Scale bar of all images: 200 μm.

Analysing the migration of cancer cells is an important goal for establishing cancer models, and HMEpiC cells with RFP and MDA-MB-231 cells with GFP were loaded into microfluidic devices for the co-culture and the observation of cancer cells migration. We began timing when the cells were adherent and the co-culture of the two types of cells was established. Then, on days 1, 2 and 3, we observed the migration of the MDA-MB-231 cells in the co-culture chambers of the mild, moderate and severe cancer models, respectively, as shown in Fig. 4. The microchannel arrays are indicated by the white dotted wireframe, the blue dotted lines show the same microchannel of each cancer model at three time points, the cells in the red circle of each cancer model are the same cell at three time points, and the red arrows indicate the migration direction. Migration of the cancer cells was clearly observed. We observed that the severe cancer model had the most migrated cells and that the mild cancer model had the fewest migration cells in the microchannel arrays. We also observed the dynamic process of cell migration in which the cancer first stretched out a filamentous protuberance into the channel, then the cell sap flew forward, and finally the back part of the cell shrank. This model was used for the subsequent quantitative analysis of the migration ability of cancer cells.

**Figure 5:**
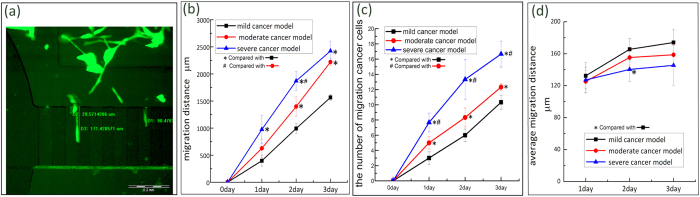
(**a**) Measurement of cancer cells migration distance, Scale bar: 200 μm. (**b**) The total migration distance of cancer cells. (**c**) The number of cancer cells which migrated into the microchannels. (**d**) The average migration distance. The error bars represent the standard deviations calculated from separate assays (n = 4); *p < 0.05, ^#^p < 0.05.

We performed 4 groups of experiments under the same conditions to study and quantify the migration of cancer cells. Based on the migration images we observed, we manually measured the number of cancer cells that migrated into the microchannel arrays and the migration distance of the MDA-MB-231 cells and then the measured data were analysed through statistical methods. The optical images of cell migration were processed (Fig. 5(a)) to measure the migration distance of the cancer cells in the microchannel arrays using Image-Pro Plus 6.0 software. The migration distance in the statistical analyses represents the migration ability of a cancer cell colony, and the average migration distance represents the average migration ability of a single cancer cell that migrated into the microchannels. We found that with increasing time, the total migration distance of the cancer cells increased (Fig. 5(b)) and that the number of cells that migrated into the microchannels increased (Fig. 5(c)). For the different cancer models, when the density of the cancer cells was higher, the total migration distance of the cancer cells was longer. Then, we analysed the average migration distance per metastatic cell within 1 day, 2 days and 3 days. As shown in Fig. 5(d), We observed a tendency that the cancer models with a higher density of HMEpiC cells exhibited a greater average migration distance. In each cancer model, with increased co-culture time, the average migration distance of the migrating cells increased rapidly within 1 day and eventually stabilised.

### The interaction analysis between normal breast cells and breast cancer cells

**Figure 6:**
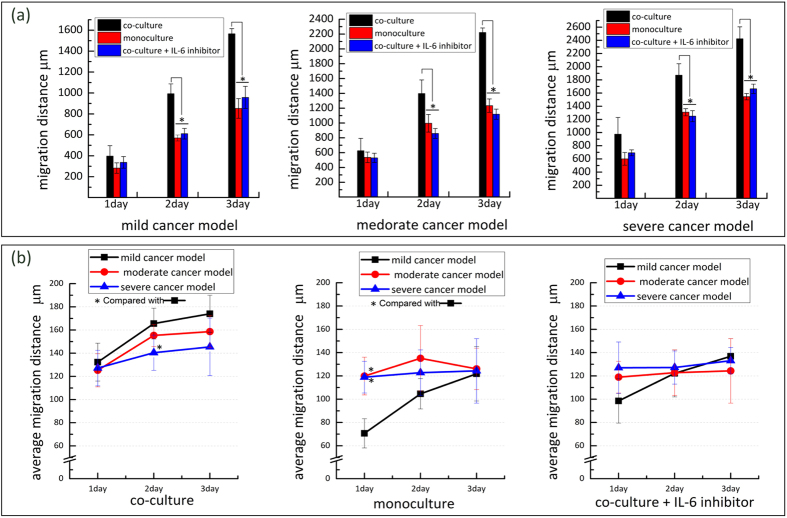
(**a**) The total migration distance of MDA-MB-231 cells under different culture conditions. (**b**) The average migration distance under different culture conditions. The error bars stand for the standard deviations calculated from separate assays (n = 3); *p < 0.05.

To investigate the interaction between normal breast cells and breast cancer cells, we solely loaded the MDA-MB-231 cells on a chip to analyse the migration of cancer cells without co-culture. We observed that the migration ability of the MDA-MB-231 cells under the monoculture condition significantly decreased (p < 0.05) compared with the co-culture condition (Fig. 6(a)). This indicates that co-culture with normal breast cells markedly aided in increasing the migration ability of the MDA-MB-231 cells. By comparing the average migration distance of the cancer cells in the two cells culture conditions, we found that the average migration distance of the cancer cells of the three cancer models decreased to different degrees under monoculture condition (Fig. 6(b)). In the monoculture condition, we did not observe the same tendency that a higher density of HMEpiC cells corresponded to greater average migration distances, which indicated the ability of HMEpiC cells to induce cancer cell migration from another perspective.

**Figure 7:**
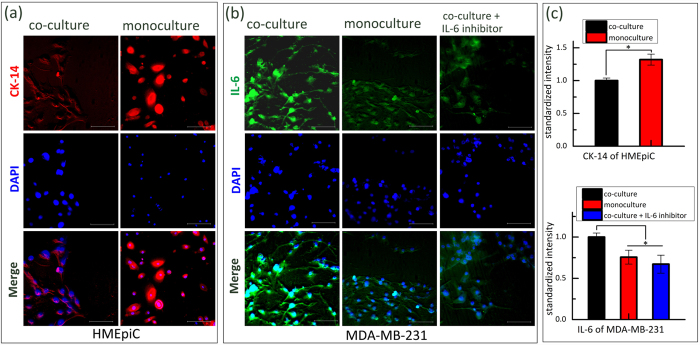
(**a**) Immunofluorescence images of CK-14 (in red). (**b**) Immunofluorescence images of IL-6 (in green). (**c**) Quantified results of the immunofluorescence images by the standardized intensity; the error bars represent the standard deviations calculated from separate assays (n = 3), (*p < 0.05). Scale bar of all images: 100 μm.

In order to assess the biological interaction of normal breast cells and breast cancer cells, we analysed the expression of CK-14, a characteristic protein of epithelial cells, on HMEpiC cells and the expression of IL-6, a characteristic protein that modulates cell migration, on MDA-MB-231 cells with or without co-culture (Fig. 7). Here, we only analysed expression of relative proteins in moderate cancer chambers because intuitively there would not be a difference in the expression levels of relative proteins among the different cancer models (Fig. S1). We found that through co-culture, CK-14 expression of HMEpiC cells significantly decreased and that IL-6 expression of MDA-MB-231 cells significantly increased (p < 0.05) (Fig. 7(c)). These results show that the enhancement of cancer cell migration ability may be relative to increasing expression of IL-6.

To verify our inference, we inhibited IL-6 expression by treating MDA-MB-231 cells with IL-6 inhibitor (IL-6 RA), and then loaded cells into chips for co-culture. By immunofluorescence staining we observed a decrease in IL-6 expression compared with the co-culture condition, and this result showed the inhibition effect of the IL-6 inhibitor (Fig. 7(b,c)). By migration tests, the decrease of migration ability of MDA-MB-231 cells compared with the co-culture condition was also observed in Fig. 6.

### Paclitaxel and tamoxifen treatments and the evaluation of anti-metastatic effect

**Figure 8:**
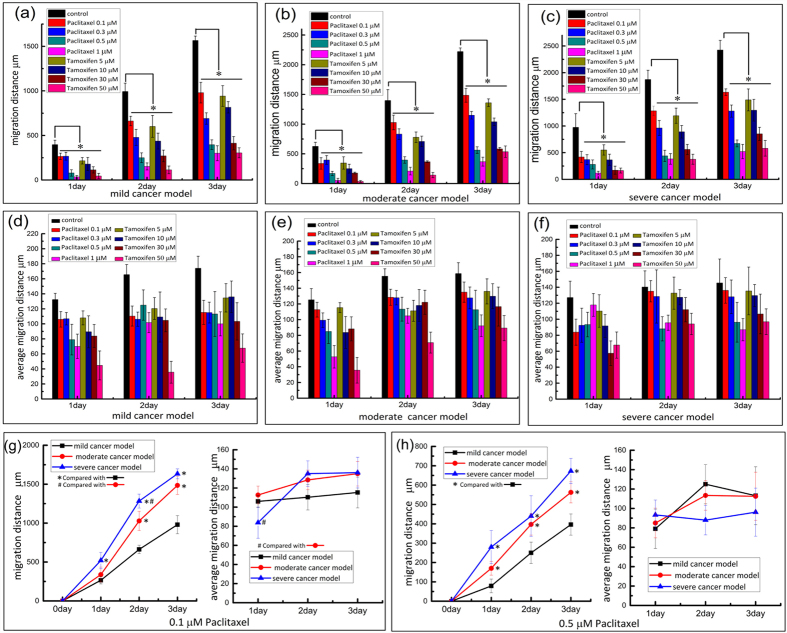
(**a**–**c**) The total migration distance of cancer cells at different concentrations of paclitaxel and tamoxifen. (**d**–**f**) The average migration distance under treatment of drugs. (**g**,**h**) Analysis of the migration ability of cancer cells treated with 0.1 μM of paclitaxel and 0.5 μM of paclitaxel respectively. The error bars represent the standard deviations in all of the charts calculated from separate assays (n = 3); *p < 0.05, ^#^p < 0.05.

To simulate a drug-screening environment to test the effect of drugs on tumours, we also utilised the co-culture of HMEpiC and MDA-MB-231 cells. Cell migration inhibition assays were carried out in the microfluidic devices. We mainly compared the effect of different concentrations of drugs on cancer cell migration through the valves based on the total migration distance and the average migration distance per metastatic cell at different time points. As shown in Fig. 8(a–c), in the mild cancer, moderate cancer and severe cancer models, we compared the total migration distance of the MDA-MB-231 cells upon treatment with different concentrations of paclitaxel and tamoxifen. Compared with the condition without drug treatments, the total migration distance of the cancer cells that processed the treatment of drugs showed a significant inhibition (p < 0.05) in the same model. There was a tendency that the migration distance of the cancer cells became shorter with increased concentrations of both paclitaxel and tamoxifen.

Then, we compared the average migration distance per metastatic cell at different concentrations of the drugs (Fig. 8(d–f)). There was a tendency that when the cells were treated with different concentrations of drugs, the average migration distance was reduced. In most cases, treatment with higher concentrations of the drugs resulted in a lower average migration distance of the MDA-MB-231 cells. We further analysed the metastatic ability of the cancer cells in the different models at the same drug concentrations, as shown in Fig. 8(g,h). We obtained the same observation that in the different cancer models, higher densities of cancer cells resulted in longer cancer cell migration distances. However, among the different cancer models, the average migration distance of the cancer cells was not significantly different.

## Discussion

Quantitation of the migratory capability at different cell densities is very important for providing a more accurate characterisation of the effects of the microenvironment^16^,^39^,^40^,^41^. Non-malignant cell components and cancer cells together participate in the building of the tumour microenvironment *in vivo*^6^,^42^. For a breast tumour, the migratory capability of cancer cells may be not the same in different regions of the tumour (Fig. 1(a)) because of different densities of cancer cells and non-malignant cells. In this study, we built a new multicellular co-culture system that mimics the tumour environment at the different regions of tumour tissue to study breast cancer cell migration. Briefly, the co-culture of HMEpiC cells and MDA-MB-231 cells was achieved to rebuild the environment of breast tumour tissue. By controlling the cell densities of the cancer cells and normal cells, mild, moderate and severe cancer models were established.

Microfluidic technology is a useful technology platform for mimicking the tumour microenvironment^43^,^44^,^45^. A microfluidic device that consisted of three co-culture chambers was designed to establish three cancer models by controlling the cell density. Although a number of co-culture cell migration studies using microfluidic devices that mimic the tumour microenvironment have been reported^11^,^27^, many of these microfluidic devices require higher operation of the cells and are difficult to operate and analyse cells in a high throughout manner. Our microfluidic chips could achieve efficient, easy manipulation and control of the cells, perform real-time monitoring of cell growth and cell migration and allow for more efficient bioassays and drug screening. For example, we designed 10-μm-high microchannel arrays (Fig. 2(c), C) to establish the controlled communication of both sides of the co-culture chambers and to observe and measure the migration of cancer cells. Both sides of the co-culture chambers spontaneously isolated the HMEpiC and MDA-MB-231 cells because of the fluid surface tension (Fig. 2(d)) through the microchannel arrays. By applying a pressure pulse in one side of the co-culture chamber, communication between the HMEpiC and MDA-MB-231 cells was achieved. The microchannel arrays contained 27 microchannels and, through imaging, we could easily confirm the position of every microchannel, monitor the migration of a certain cell and observe the dynamic migration of cancer cells (Fig. 4).

Based on live/dead assays, we proved that our microfluidic devices supported cell growth. Then, by transfecting the HMEpiC and MDA-MB-231 cells with RFP and GPF, respectively, the migration of the cancer cells could be easily detected. We observed the dynamic process of cell migration: the cancer first stretched out a cellular protrusion into the channel, then the cell sap flew forward, and finally the back part of the cell shrank (Fig. 4). Statistical analyses of the migration of MDA-MB-231 cells were performed. We found that among the three cancer models, the cancer model with a higher density often displayed more obvious cell migration and stronger migration ability (Fig. 5(b,c)). The cancer model with the higher cell density of HMEpiC cells induced greater cancer cell migration (Fig. 5(d)). In the cancer model, the average migration distance of the migrating cells increased rapidly within 1 day and eventually stabilised with the passage of time, which indicated that communication between the HMEpiC and MDA-MB-231 cells was quickly established and eventually achieved a balance. These results illustrated that in our microfluidic devices, the density of the cancer cells often determined the probability of the occurrence of the metastatic cells and that the inducement of a normal cell, to a certain extent, affected the migration ability of every cancer cell. Therefore, the number of breast cancer cells is a key factor in determining the migration ability of breast cancer tumour cells; this explains that it is only after a tumour develops into a certain size that tumour metastasis occur to form a second tumour colony.

To further investigate the interaction between HMEpiC and MDA-MB-231 cells, the expression of relative proteins that determine the function or characteristics of two types of cells was analysed. We chose CK-14, a characteristic protein of epithelial cells, to analyse the effect of MDA-MB-231 cells on HMEpiC cells. The result showed that the expression of CK-14 decreased after co-culture with cancer cells. This is an important feature of EMT^46^,^47^, and we also observed a morphological change in the HMEpiC cells co-cultured with cancer cells (Fig. S2). IL-6 is a pro-inflammatory cytokine that alters cell morphology and modulates cell migration, and increased secretion of IL-6 was observed in breast cancer cells that were co-cultured with conditional medium of breast epithelial cells^36^,^48^. In our work, we also found increased expression of IL-6 in MDA-MB-231 cells and a stronger migration ability of MDA-MB-231 cells in the co-culture system compared with the monoculture condition. Upon inhibiting IL-6, the migration ability of MDA-MB-231 cells was obviously inhibited, which further indicated that increased secretion of IL-6 induced by HMEpiC cells led to the increase in the migration ability of MDA-MB-231 cells.

We used paclitaxel and tamoxifen to act on the cancer models and to explore the effect of drugs on cell migration. After the addition of a drug, the migration ability of the cancer cells was significantly inhibited (p < 0.05). We observed a trend that with increased drug concentration, the ability of the drug to inhibit migration was strengthened. Among the three cancer models treated with the same drug concentration, when the cancer cell densities were higher, there was a stronger metastatic ability. We analysed the mechanisms of the drugs’ inhibition and identified two main reasons for the effect of the drugs on the migration of the cancer cells. First, the death of the cells led directly to a reduction in the number of cancer cells and normal cells and directly led to the decreased migration ability of the cancer cells (Fig. S3). Liu *et al*. demonstrated that there was an internal connection between cellular protrusion formation and the metastatic ability of cancer cells^49^. Thus, the other reason may be due to the effect of the drugs on the cell morphology of the MDA-MB-231 cells, which was altered and did not benefit cell migration. Using microfluidic devices, we observed that there was a significant decrease in the cellular protrusion of the cancer cells when the MDA-MB-231 cells were treated with the drugs (Fig. S4).

## Conclusion

In summary, a microfluidic co-culture system that mimicked different regions of a metastatic breast tumour was successfully established to study cancer cell migration and anti-cancer drug screening. HMEpiC cells and MDA-MB–231 cells were co-cultured to establish mild, moderate and severe cancer models. Using this platform, it was possible to monitor not only cell vitality in real time but also cancer cell migration by transfecting the HMEpiC and MDA-MB–231 cells with RFP and GFP, respectively. We found that the density of the cancer cells often determines the probability of the occurrence of a metastatic cell and that the induction of normal cells, to a certain extent, affects the velocity of each cancer cell. The results showed that the increased migration ability of MDA-MB-231 cells co-cultured with HMEpiC cells was relative to the increased secretion of IL-6 and that this was verified via an IL-6 inhibitor assay. In addition, this co-culture also led to decreased CK-14 secretion and morphological changes in the HMEpiC cells. Finally, by adding different concentrations of paclitaxel and tamoxifen, we observed a significant migration inhibition of the MDA-MB–231 cells. This microfluidic system provides a novel way to mimic an *in vivo* tumour microenvironment, which could be used for the quantitation of migratory capability and anti-metastatic drug screening.

## Additional Information

**How to cite this article**: Mi, S. *et al*. Microfluidic co-culture system for cancer migratory analysis and anti-metastatic drugs screening. *Sci. Rep.* **6**, 35544; doi: 10.1038/srep35544 (2016).

## Supplementary Material

Supplementary Information
