# Supplementary material for: Microfluidic co-culture system for cancer migratory analysis and anti-metastatic drugs screening
Source: Sci Rep. 2016 Oct 20;6:35544. doi: 10.1038/srep35544 (PMC5071858; doi:10.1038/srep35544)
Supplement: Supplementary Information [file srep35544-s1.pdf]

Supplementary information for

Microfluidic co-culture system for cancer migratory analysis and anti-metastatic drugs screening

Shengli Mi,[a,e+] Zhichang Du,[a+] Yuanyuan Xu,[a] Zhengjie Wu,[a] Xiang Qian,[a] Min Zhang,[a] Wei Sun[a,b,c,d*]

[a.] *Graduate School at Shenzhen, Tsinghua University, Shenzhen, P. R. China. E-mail: weisun@mail.tsinghua.edu.cn; Tel: +8675526036329; Fax: 8675526036356*

[b.] *Department of Mechanical Engineering and Mechanics, Tsinghua University, Beijing, China.*

[c.] *Department of Mechanical Engineering, Drexel University, Philadelphia, PA, USA.*

[d.] *Tsinghua-Berkeley Shenzhen Institute, Shenzhen, P. R. China.*

[e.] *Open FIESTA Center, Tsinghua University, Shenzhen 518055, PR China*

[+]*These authors contributed equally to this work.*

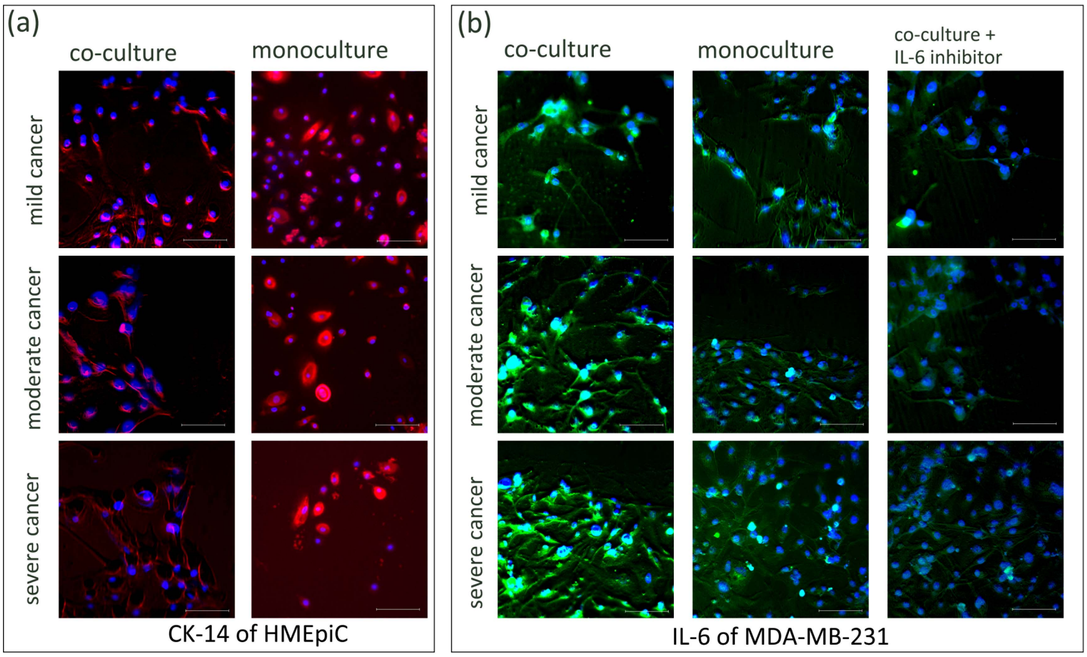

Supplementary Fig. S1. (a) Immunofluorescence images of CK-14 (in red) of HMEpiC cells under co-culture and monoculture conditions in three cancer models. (b) Immunofluorescence images of IL-6 (in green) of MDA-MB-231 cells under co-culture, monoculture and IL-6 inhibitor conditions in three cancer models. All of the optical images have the same scale bar: 100 µm, the nucleus is in blue.

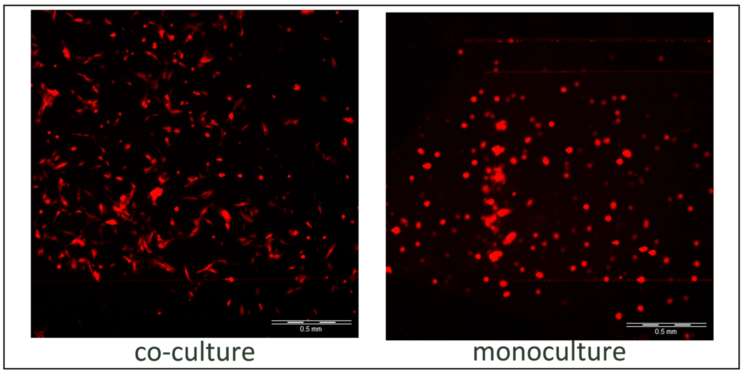

Supplementary Fig. S2. Morphological characteristics of HMEpiC cells in co-culture condition and monoculture condition. HMEpiC cells in red were transfected with RFP and cultured in moderate cancer model. The optical images have the same scale bar: 500 µm.

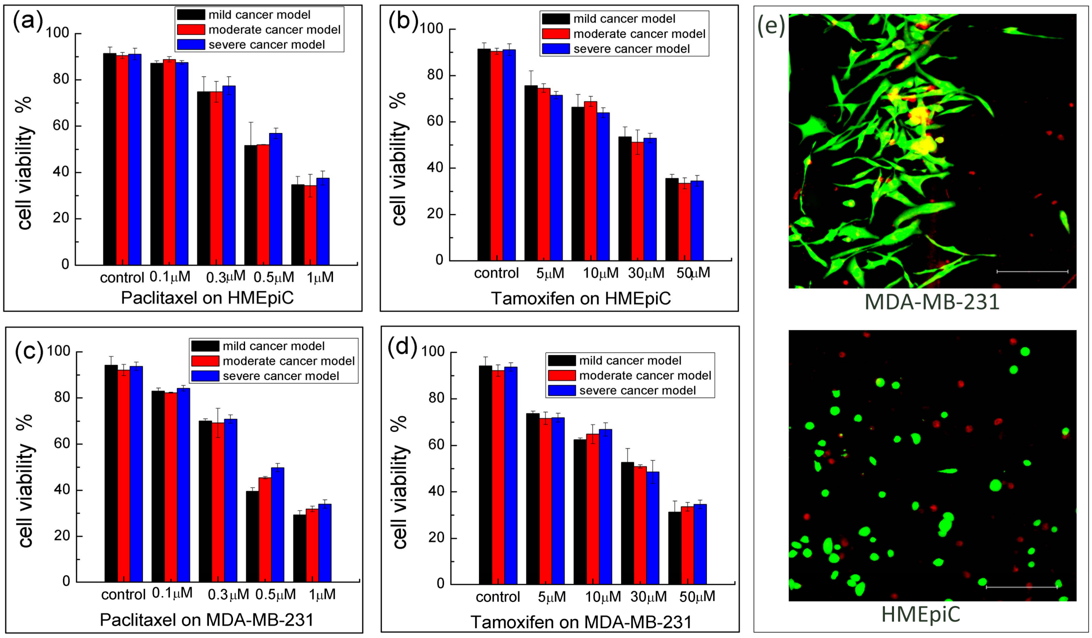

Supplementary Fig. S3. (a-d) Statistical analysis of the cell viability of the MDA-MB-231 or HMEpiC cells at different concentrations of paclitaxel or tamoxifen, respectively. The error bars stand for the standard deviations calculated from separate assays (n=4). (e) The merged images of the live/dead staining images of the MDA-MB-231 and HMEpiC cells. The sample of images was at 10 μM tamoxifen in the moderate cancer model. The optical images have the same scale bar: 200 μm.

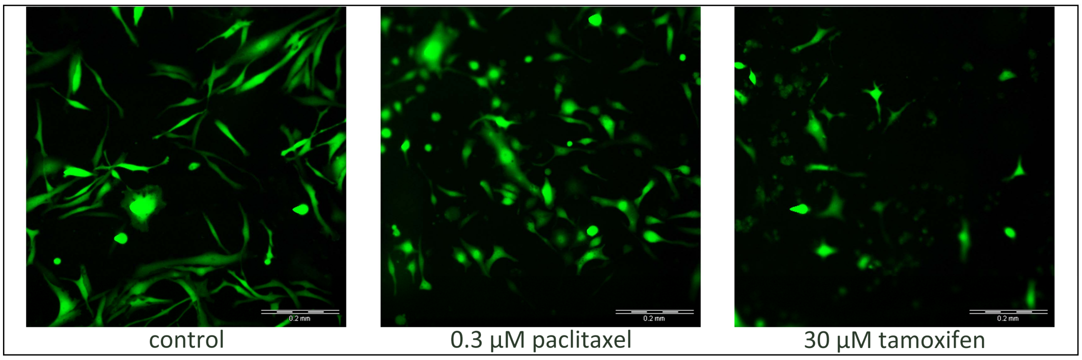

Supplementary Fig. S4. Morphological characteristics of MDA-MB-231 without or with anti-cancer drugs treatment. MDA-MB-231 cells in green were transfected with GFP and cultured in moderate cancer model. The optical images have the same scale bar: 200 μm.
